# The omega-3 index in Alzheimer's disease: Ready for prime time?

**DOI:** 10.1093/ajcn/nqac248

**Published:** 2022-10-17

**Authors:** Hussein N Yassine

**Affiliations:** Department of Medicine, Keck School of Medicine, University of Southern California, Los Angeles, CA, USA; Department of Neurology, Keck School of Medicine, University of Southern California, Los Angeles, CA, USA

See corresponding article on page 1492.

The omega-3 (ω-3) index is the sum of the percentages of EPA and DHA from the total fatty acids in blood. Over the past 2 decades, some of the major observational cohorts, including the Women's Health Initiative Memory Study (1), Framingham Study (2), and the 3-City Study (3), reported associations between the lowest quartile of the ω-3 index and worse cognitive measures, brain MRI measurements, and greater risks of dementia. These reports raised the prospect that the ω-3 index could be used to identify persons at risk of dementia and hypothesized that raising the ω-3 index may lower the rate of cognitive decline. To date, findings from trials investigating the effects of ω-3 supplementation on cognitive outcomes have not been compelling (4). The Multidomain Alzheimer Preventive Trial included an ω-3 supplementation arm with 800 mg of DHA and 225 mg of EPA consumed daily over 3 years but did not result in any meaningful cognitive benefit (5). A subanalysis of the Multidomain Alzheimer Preventive Trial cohort revealed an association between the lower quartile of the ω-3 index and greater cognitive decline in the placebo group (6). A question remained of whether selectively targeting those in the lowest ω-3 index with ω-3 supplements could result in cognitive benefits.

In this issue of *The American Journal of Clinical Nutrition*, Rouch et al (7) analyzed the cross-sectional and retrospective longitudinal associations between scores on the erythrocyte ω-3 index and cognition, brain imaging, and biomarkers among older adults in samples from 832 individuals who participated in the Alzheimer's Disease Neuroimaging Initiative (ADNI). A low ω-3 index score was not associated with cognition, hippocampal or increased white matter hyperintensity (WMH) volumes, or brain Positron Emission Tomography (PET) Aβ or tau values after adjustment for demographics. However, a low ω-3 index score was associated with greater Aβ accumulation in the retrospective analysis and with lower performance on the Wechsler Memory Scale and higher tau accumulation among apoE ε4 carriers compared to noncarriers in the cross-sectional analysis. A limitation of this study is that the ADNI cohort largely consisted of a generally healthy, white, educated, and older group (55–90 years), which may also be too old to detect the earliest stages of disease. In addition, individuals with a high modified Hatchinski Ischemic Scale score (8), a measure of vascular risk factors, were excluded.

These findings from the ADNI raise questions on the utility of the ω-3 index for identifying persons at greater risk of cognitive decline in an educated, older, white population with lower vascular risk factors. While a low ω-3 index score or fish consumption are risk factors for dementia, the risk conferred by a low ω-3 index score might be offset by other factors. For example, in the Adventist population, 1 of the 5 original “blue zones” (9) in the world with a high level of education, healthy lifestyles, and high longevity, many individuals follow a predominantly vegetarian dietary pattern that excludes fish consumption (10) and, presumably, they have low ω-3 index scores. Vegetarians who do not consume fish did not perform worse on cognitive measures compared with matched controls who consumed fish (11). Emerging research supports the importance of complex dietary patterns, such as the Mediterranean diet, as better predictors of cognitive performance than any single dietary component (12). Accordingly, a lower ω-3 index score in a background of a poor lifestyle and other dementia risk factors may better predict dementia progression, while a lower index score in an educated group with healthy lifestyles and lower vascular risk factors may be less informative.

Carrying the apoE ε4 is the strongest genetic risk factor for late-onset Alzheimer's disease. There is some evidence indicating an association of low ω-3 index scores in apoE ε4 carriers with greater cognitive decline (13). Decades before the onset of dementia, apoE ε4 is associated with greater DHA brain uptake using ^11^C DHA PET scans, suggesting a greater brain utilization of plasma-derived DHA and implying vulnerability to lower DHA consumption (14). Indeed, cognitively normal and younger ApoE ε4 carriers who consume more seafood have a lower risk of Alzheimer's disease than age matched non-carriers (15). In an era of personalized nutrition, the ω-3 index may serve a better role in predicting dementia risks in select populations at risk of Alzheimer's disease than the general population. Whether increasing the ω-3 index scores by ω-3 supplements in this group will translate into cognitive benefits is yet to be determined.

## Notes

HNY is supported by the Kenneth and Bette Volk Endowed Chair of Neurology at University of Southern California, and grants from the National Institute on Aging (RF1AG076124, R01AG055770, R01AG067063, R01AG054434, R21AG056518, and P30AG066530); the Alzheimer's Drug Discovery Foundation (GC-201711-2014197), and from generous donations from the Vranos and Tiny Foundations and Ms Lynne Nauss.

Author disclosures: The author reports no conflicts of interest.
